# A Brief Web-Based Nutrition Intervention for Young Adult University Students: Development and Evaluation Protocol Using the PRECEDE-PROCEED Model

**DOI:** 10.2196/11992

**Published:** 2019-03-28

**Authors:** Megan Whatnall, Amanda Patterson, Melinda Hutchesson

**Affiliations:** 1 School of Health Sciences, Faculty of Health and Medicine University of Newcastle Callaghan Australia; 2 Priority Research Centre for Physical Activity and Nutrition University of Newcastle Callaghan Australia

**Keywords:** eHealth, young adults, universities, students, diet

## Abstract

**Background:**

Young adults are a priority population for nutrition interventions because of the high prevalence of unhealthy eating behaviors, high risk of weight gain, and the importance of this life stage for developing lifelong eating behaviors. Innovative intervention strategies are needed to reach and engage young adults, whereas more detailed reporting of intervention development and testing would facilitate progress in this challenging research area.

**Objective:**

This paper describes the development of the EATS (Eating Advice To Students) intervention, a targeted, brief Web-based nutrition intervention for young adult (17 to 35 years) university students, and describes the pilot randomized controlled trial (RCT) to assess intervention feasibility.

**Methods:**

EATS was developed using the PRECEDE-PROCEED model. The development involved a cross-sectional survey of university students’ eating behaviors and determinants, a systematic review of brief nutrition interventions, and consultation with a project steering committee. EATS was developed as a website with 4 components: (1) brief screening quiz with personalized feedback, (2) provision of information, tips, and strategies for each target eating behavior (consumption of vegetables, fruit, discretionary foods, and breakfast) and 2 guided exercises to facilitate behavior change, (3) goal setting, and (4) creating strategies. A pilot RCT with students from the University of Newcastle, Australia, was conducted from February to July 2018. The students were randomized to EATS or a brief Web-based alcohol intervention (attention control). The process evaluation included intervention acceptability (Web-based survey postintervention completion) and objective usage data (collected in real time). Efficacy data (Web-based survey at baseline and 3 months) included diet quality, consumption of target food groups (eg, fruits and vegetables), alcohol intake, self-efficacy to perform target eating behaviors, and well-being.

**Results:**

Collection of the 3-month follow-up data was completed in July 2018.

**Conclusions:**

EATS presents an innovative solution to many of the difficulties faced in targeting young adults to improve their eating behaviors. Given the strong methodological approach undertaken, this study provides a significant contribution to advance this research area.

**Trial Registration:**

Australian New Zealand Clinical Trials Registry ACTRN12618000118202; https://www.anzctr.org.au/Trial/Registration/TrialReview.aspx?id=374365&isReview=true (Archived by WebCite at http://www.webcitation.org/765o5fVwa)

**International Registered Report Identifier (IRRID):**

DERR1-10.2196/11992

## Introduction

### Background

Young adults (17 to 35 years) are a priority population for nutrition interventions because of their high prevalence of unhealthy eating behaviors [1,2], high risk of weight gain [3,4], and the importance of this life stage for developing lifelong eating behaviors [5]. However, reaching and engaging young adults in health behavior change interventions, including nutrition interventions, is challenging [6,7]. Reaching and engaging young adults may be challenging because of factors such as transient living arrangements and perceptions such as seeing health behavior interventions as irrelevant to their life stage, and giving their health a low priority compared with other time commitments [8,9]. The need for interventions targeted to young adults to address poor health behaviors among this group has been recognized, and therefore, this area of research is now gaining momentum [10-12]. For example, in a recent review of lifestyle interventions aiming to prevent weight gain in young adults, 19 of the 30 included studies had a nutrition component and dietary outcomes [12]. However, there is still further research to be done, with higher quality and detail in reporting [13].

One avenue for further exploration is targeting young adults through the university setting [14]. This is because the number of individuals across the globe enrolling in universities is increasing, with many in the young adult age category. For example, upward of 20 million students are enrolled in universities in the United States and Europe [15,16], with 40% of those in the United States aged 18 to 24 years [15]. In Australia, approximately 60% of the 1.5 million university students are aged between 18 and 24 years [17]. Furthermore, university initiatives to promote optimal health are gaining traction internationally as the utility of this setting to support health promotion interventions because of its existing infrastructure (eg, technology, facilities, and expert researchers and health professionals within the university staff) is increasingly recognized [18]. Furthermore, young adults may be more amenable to intervention and behavior change while immersed in the university learning environment [19], as well as being in a formative stage for developing lifestyle behaviors [14,20].

Developing appealing and engaging interventions also requires cognizance of the many factors individuals face in this life stage because of their impact on behavior and behavior change, for example, changes in employment, tertiary study, and family and social life, as well as development in terms of self-identity and self-efficacy. One of the key barriers to healthy eating for young adults is lack of time [9], and with this in mind, exploring intervention approaches that cater to this (ie, brief interventions) could be worthwhile. On this note, it has been acknowledged that the use of community-based participatory research models to guide intervention development can contribute to more engaging and effective health behavior interventions [8,21]. In the current context, the university setting provides an ideal platform for participatory research.

### Objectives

This paper describes the development and evaluation plan for a targeted, brief Web-based nutrition intervention for young adult (17-35 years) university students using the PRECEDE-PROCEED model.

## Methods

### Overview

**Figure 1:**
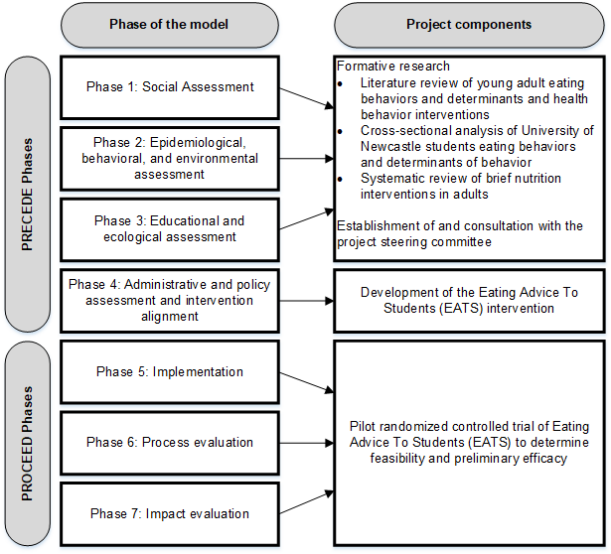
Overview of development and evaluation of the Eating Advice To Students (EATS) intervention for young adult university students, by phase of the PRECEDE-PROCEED model.

The PRECEDE-PROCEED planning model developed by Green et al [22] was used as the basis for the development and evaluation of the Eating Advice To Students (EATS) intervention. This model is an ecological approach to health promotion, and it has been used extensively in previous applications of health promotion interventions, including in young adults and university students [8,23]. The model comprises 8 phases, which can be broadly categorized into planning (PRECEDE, phases 1-4) and evaluation (PROCEED, phases 5-8) [24]. An overview of the PRECEDE-PROCEED model and a description of how each component of this project relates to the model is provided in Figure 1 and outlined below. As this is a pilot study, outcome evaluation (phase 8) was not assessed.

#### Phase 1: Social Assessment

To develop an intervention that is acceptable and appropriate, the focus of phase 1 was to develop an understanding of the target population, specifically the University of Newcastle (UON) student population, and to explore its demographics, social norms, and health problems [22]. A literature review was undertaken and a steering committee, including members of the target population and key staff in the university setting, was formed. The literature review highlighted the difficulty in reaching and engaging young adults to change their lifestyle behaviors [13]. It was also highlighted that undertaking university study is a key experience for many young adults [15,16] and that unhealthy eating behaviors are also characteristic of young adults who attend university [25,26]. It was based on these findings that the target population was further refined to include young adult (17 to 35 years) university students. The steering committee was also formed during this phase, with its role being to guide intervention development, assist in piloting the intervention, and to have ongoing involvement in ensuring translation into University Health Promotion. The steering committee includes key staff members (eg, from University Health Promotion, Student Residences, and Student Communications and Marketing) and a diverse group of students representing undergraduate and postgraduate, health and nonhealth degree background, international and domestic, and male and female.

#### Phase 2: Epidemiological, Behavioral, and Environmental Assessment

Phase 2 was focused on developing a logic model of the health problem, linking the health problem to environmental and behavioral determinants [22]. During this phase, the goal was to identify the priority eating behaviors and determinants to be targeted and addressed in the intervention. To achieve this, the literature was reviewed and a cross-sectional analysis of the data from the UON Student Healthy Lifestyle Survey (SHLS) 2016 was conducted. From the literature, it was identified that students typically have low consumption of nutrient-rich foods, particularly fruits and vegetables [27], and high consumption of energy-dense, nutrient-poor foods, especially takeaway foods and confectionary [28,29]. In addition, the major determinants influencing eating behaviors were found to be environmental factors such as living situation, social environment, financial status, as well as personal factors such as age and gender [30-34]. Gaps in the evidence base included that there are few studies among university students that explore a broad range of eating behaviors and the associations among them, as well as a broad range of determinants. Furthermore, there is a distinct lack of studies of this nature in Australian students. The cross-sectional analysis of the SHLS included 4383 students (70.59% [3094/4383] female; mean age 25.5 (SD 9.3) years, and 92.01% [4033/4383] domestic students). Eating behaviors were assessed using short diet questions relating to consumption of fruits, vegetables, breads, cereals, red meat, a range of discretionary (ie, energy-dense and nutrient-poor) food categories (eg, confectionary, processed meat products, and hot chips, wedges or fried potatoes), and breakfast [35]. The priority eating behaviors identified were fruits (45.74% [2005/4383] consuming <2 servings per day), vegetables (91.31% [4002/4383] consuming <5 servings per day), discretionary foods (eg, 68.67% [3010/4383] consuming confectionary 1-2 times per week or more; 54.55% [2391/4383] consuming hot chips, wedges or fried potatoes 1-2 times per week or more), and breakfast (41.75% [1830/4383] not consuming daily). The key determinants identified were living situation, gender, and faculty of study, with females, students living in their own home or on-campus, and students enrolled in the Faculty of Health and Medicine found to have healthier eating behaviors overall [36].

#### Phase 3: Educational and Ecological Assessment

Phase 3 builds on the logic model from phase 2 by exploring the predisposing, reinforcing, and enabling factors influencing behavior [22]. To explore these, the literature was reviewed, a systematic review was conducted to evaluate the effectiveness of a potentially applicable intervention approach, and the steering committee was consulted for input. From the literature review, key predisposing factors for healthy eating were desirability for improved health outcomes, weight management, and attractiveness, whereas the main deterrent was lack of motivation [9,37-39]. The key reinforcing factors were having positive social support and social influence [9,37-39]. Enabling factors, that is, conditions facilitating or preventing healthy eating, were in terms of having the time, facilities or skills to prepare healthy foods, the relative low cost and high availability of unhealthy food options, and social expectations to consume unhealthy foods in certain situations [9,37-39]. With time being a major barrier to healthy eating and to participation in health behavior interventions, the potential efficacy of a brief intervention was explored. To add precedent, brief interventions have been found to be effective in reducing alcohol intake among young adults and university students [20,40]. As the second step within this phase, a systematic review was conducted with the aim of evaluating the effectiveness of brief (ie, single session) nutrition interventions in adults and to identify behavior change techniques (BCTs) associated with effective interventions [41]. The review specifically included studies in all adults (ie, ≥18 years) rather than young adults so as not to limit the number of included studies and the scope of the review. This is because no systematic reviews had been conducted assessing brief or single-session nutrition interventions. The participant characteristics of the included studies suggest it is appropriate to translate the findings to the young adult population, for example, 20 of the 45 included studies had a participant mean age between 18 and 35 years, although the majority of these were not targeted to a young adult population. It was found that brief interventions, particularly those that were tailored and instructional, can improve dietary behaviors in the shorter term. BCTs identified as effective were incorporated in the development of EATS. Finally, a summary of the findings and proposed intervention content from all formative research undertaken in phases 1 to 3 was presented to the steering committee members and a meeting was held to discuss and gather their feedback.

#### Phase 4: Administrative and Policy Assessment and Intervention Alignment

**Figure 2:**
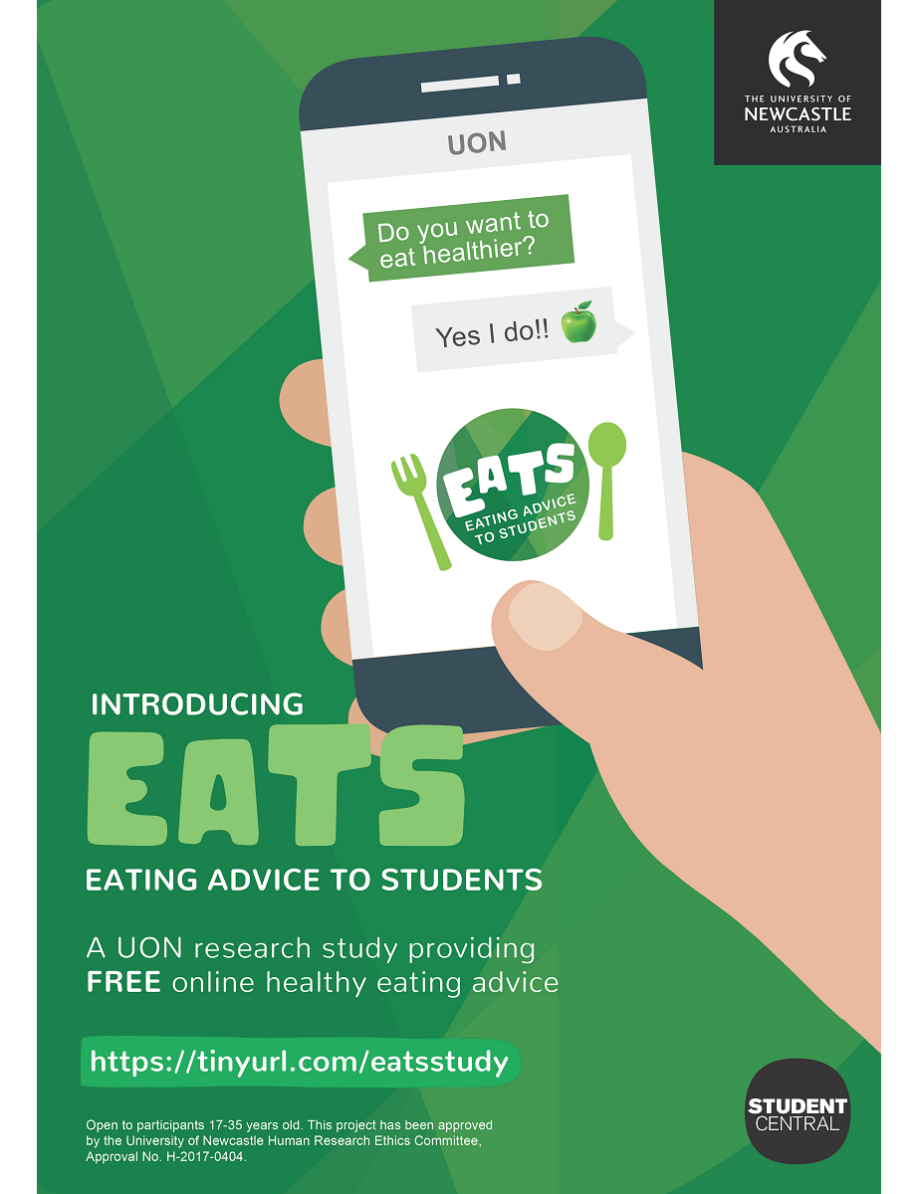
Sample recruitment poster used to recruit young adult university students to the Eating Advice To Students (EATS) brief Web-based nutrition intervention pilot randomized controlled trial.

**Figure 3:**
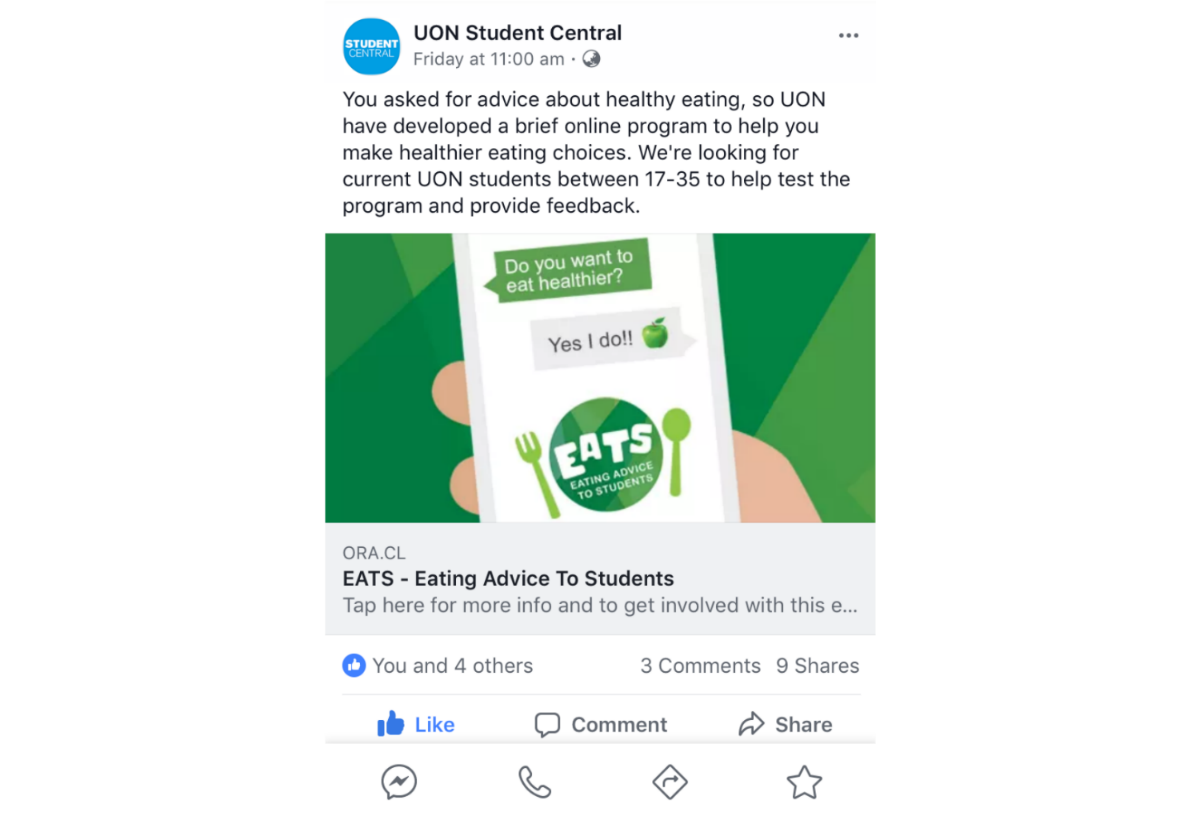
Sample Facebook post used to recruit young adult university students to the Eating Advice To Students (EATS) brief Web-based nutrition intervention pilot randomized controlled trial.

Phase 4 focuses on determining the program and intervention components required to effect the desired behavior changes, with respect to the determinants of behavior identified in the previous phases and with consideration to the organizational, policy, and administrative resources available [22]. This phase requires the inclusion of behavior change theories to support the translation of the knowledge gained from previous phases, that is, the causes and determinants of behavior, into the behavior change the intervention aims to achieve. During this phase, (1) the EATS intervention was developed, including the program logo and recruitment materials as shown in Figures 2 and 3 and (2) both EATS and all design materials were pretested and evaluated with the steering committee.

#### Development of Eating Advice To Students

EATS was developed based on the findings from the previous phases as well as by drawing on the social cognitive theory (SCT) [42], the theory of planned behavior (TPB) [43], best practice guidelines (ie, the Australian Dietary Guidelines) [44], and the experience and expertise of the research team. The SCT and TPB were chosen as their constructs reflect key factors influencing behavior and behavior change in the target group, such as social environment and social norms, and because of their previous applications in efficacious health behavior interventions in young adults [8,45,46]. EATS was developed as a brief or single-use intervention. The goal of EATS is to facilitate improvement in overall diet quality, including targeting 4 specific eating behaviors: consumption of fruits, vegetables, discretionary foods (ie, foods that are not necessary for a healthy diet as they are energy-dense and high in saturated fat, added sugars, added salt or alcohol, and low in fiber such as confectionary, hot chips, and sugar-sweetened beverages) [44], and breakfast. The intervention involves password-protected access to the EATS website with instructions to access on a single occasion. The website includes 4 components: (1) a brief screening quiz providing personalized feedback on eating behaviors and barriers to healthy eating; (2) provision of information, tips, and strategies for each target behavior and 2 guided exercises to facilitate behavior change; (3) goal setting; and (4) creating strategies. For the goal setting and creating strategies components, participants could select goals and strategies from the examples provided, write their own using the instructions provided, or both. The website directs participants to complete the components in that order, with participants specifically encouraged to complete components 2 to 4 for those eating behaviors identified in component 1 as being outside the dietary guidelines. The website also provides links and resources for further information, for example, a cookbook with cheap and healthy recipe ideas. The estimated completion time for the intervention is approximately 25 min depending on the number of components completed and resources accessed, with components 1, 3, and 4 estimated to take up to 5 min each and component 2 estimated to take up to 10 min. The key determinants identified from the previous phases were incorporated in the intervention content, for example, different living situations were considered in the examples and advice provided in terms of facilities available for food storage and preparation, lack of time was considered in terms of providing quick and easy meal ideas, and age and gender were considered in the visual design of the website. The intervention components were mapped to BCTs based on the 93-item Behavior Change Taxonomy v1 by Michie et al [47] and SCT and TPB constructs [42,43], as detailed in Table 1. Behavior change theories and BCTs have been used in combination as the theory provides the basis to explain behavior change, whereas the BCTs are the specific, practical components used to change behavior [48].

**Table 1:** Behavior change techniques and theory constructs applied in the Eating Advice To Students (EATS) brief Web-based nutrition intervention, by intervention component.

Intervention component	Behavior change techniques	Behavior change theory constructs
Screening quiz with personalized feedback on eating behaviors and barriers to healthy eating	Feedback on behavior, social support (emotional), information about health consequences, credible source, restructuring the social environment, identification of self as role model, and framing/reframing	TPB^a^: Attitude (eg, participants advised to reframe their thinking around achieving a balanced diet); SCT^b^: Knowledge (eg, providing information on Australian Dietary Guidelines)
Information, tips, and strategies for each target behavior	Instruction on how to perform the behavior, information about health consequences, information about social and environmental consequences, salience of consequences, demonstration of the behavior, social comparison, credible source, framing/reframing, restructuring the physical environment, restructuring the social environment, and avoidance/reducing exposure to cues for the behavior	SCT: Outcome expectancies (eg, providing information on health risks and benefits)
Goal setting	Goal setting (behavior), information about antecedents, instruction on how to perform the behavior, prompts/cues, restructuring the physical environment, restructuring the social environment, avoidance/reducing exposure to cues for the behavior, and focus on past success	SCT: Goals (participants instructed in setting goals and plans to achieve them); TPB: Perceived behavioral control and SCT: Perceived self-efficacy (eg, participants encouraged to focus on past successful behavior when setting goals/plans)
Creating strategies	Problem solving, action planning, information about antecedents, instruction on how to perform the behavior, prompts/cues, behavioral practice/rehearsal, behavior substitution, habit formation, habit reversal, restructuring the physical environment, restructuring the social environment, avoidance/reducing exposure to cues for the behavior, and mental rehearsal of successful performance	TPB: Perceived behavioral control and SCT: Perceived facilitators and impediments (eg, participants encouraged to plan for situations where achieving healthy eating is difficult)

^a^TPB: theory of planned behavior.

^b^SCT: social cognitive theory.

The UON Student Communications and Marketing team was engaged to develop a logo for EATS and recruitment materials including a poster, digital signage, and social media posts (Facebook and Twitter). Sample recruitment materials are shown in Figures 2 and 3. Key points included in the brief, based on the findings from the previous phases, were to ensure the materials were targeted toward 17 to 35 year old young adults, equally appealing to both genders, and emphasizing particular characteristics of EATS, for example, brief and Web-based.

#### Pretesting

The project steering committee was consulted to pretest and provide feedback on the beta version of the EATS intervention, including content and functionality, and to evaluate and provide feedback on the logo and recruitment materials. The feedback and suggestions were incorporated into the final versions of both.

#### Phases 5 to 7: Implementation, Process, and Impact Evaluation

Phase 5, implementation, involves finalizing the implementation and evaluation plans and support of program delivery before implementing the program. Phase 6, process evaluation, assesses program acceptability and usage, as well as recruitment and retention (primary outcomes). Phase 7, impact evaluation, assesses change in behavior or determinants of behavior [22]. Diet quality is a primary outcome, whereas all others are secondary outcomes. The plans for process and impact evaluation are outlined below.

### Study Design

**Figure 4:**
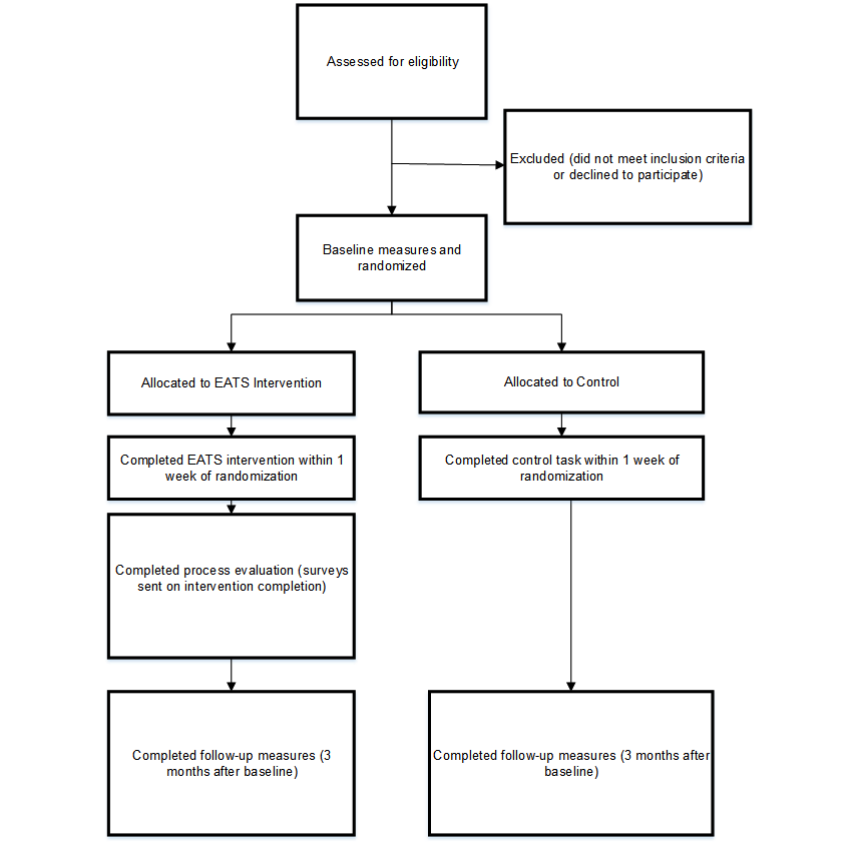
Diagrammatic summary of study timelines and flow of the Eating Advice To Students (EATS) brief Web-based nutrition intervention pilot randomized controlled trial.

The EATS intervention was evaluated in a pilot randomized controlled trial (RCT) with participants randomly allocated to the EATS intervention or attention control group from February to March 2018 and followed up from May to July 2018. Measures were collected at baseline and 3 months after baseline, with the exception of program acceptability (measured post intervention completion) and intervention usage (measured in real time). A diagram of the study timeline and flow is presented in Figure 4. Ethics approval was obtained from the UON Human Research Ethics Committee (H-2017-0404), and the trial was registered with the Australian New Zealand Clinical Trials Registry (ACTRN12618000118202p).

### Objectives and Hypotheses

The aim was to evaluate the feasibility of a brief (one-time use) Web-based nutrition intervention for young adult (17 to 35 years) university students, including process evaluation (phase 6), program acceptability and demand (usability, appropriateness, and usage) and the intervention’s ability to recruit and retain the target group and impact evaluation (phase 7), the intervention's impact on eating behaviors 3 months later compared with an attention control group who completed a brief Web-based alcohol intervention. It was hypothesized that EATS would have high acceptability among young adult university students. It was also hypothesized that young adult university students who completed EATS would have greater improvements in eating behaviors 3 months later compared with those who completed the control intervention.

### Participants and Recruitment

Students aged 17 to 35 years from the UON, Australia, were recruited for the pilot study. Exclusion criteria included having a medical condition requiring a prescribed diet (eg, diabetes). The participants were recruited via posts on the University social media pages (Facebook and Twitter) and posters and digital signage displayed across University campuses. The recruitment was also supported by the project steering committee that further promoted the study via personal networks within the university. Participants were reimbursed for their time, including a gift voucher to the value of Aus $10 after completing baseline measures and a second gift voucher to the value of Aus $10 after completing follow-up measures. All measures, including baseline, follow-up, and process evaluation, were collected via Web-based surveys conducted using Qualtrics Survey Platform (Qualtrics).

#### Sample Size

As this is a pilot study, a formal sample size calculation was not performed. The total sample size was set at 126 (ie, 63 per group). This would exceed the median sample size of 30.5 among nondrug trials in a review of the methods and conduct of pilot and feasibility trials [49], ideally allowing for more diversity within the sample in terms of sociodemographics and student-related characteristics, and this was also based on the time available for recruitment to allow the study to be conducted within the university semester (5 weeks), as well as study funding. As males are often underrepresented in health behavior research, a lower limit of 30% of the target sample size being male was set, which would exceed the proportion of males typically achieved in previous intervention studies among this target group [7]. To maximize the number of male participants enrolled, interested females were waitlisted once the proportion of females reached 70% of the target sample size. As the proportion of 30% male participants was unable to be reached, female participants were invited from the waitlist until the recruitment period was complete.

#### Randomization

Randomization was conducted by an external statistician. Allocation was stratified by gender (female; male and other gender identity). Allocation sequences within strata were generated by a statistical software program, using permuted block randomization with random block sizes of 2 and 4.

#### Control Group

Attention control group participants completed Thrive, a brief Web-based intervention designed for university students with a focus on alcohol intake [50]. Thrive provides feedback on their current alcohol intake and related risk, as well as links to further resources for support. The control group participants also received access to the EATS intervention after completing follow-up measures.

### Measures

#### Process Evaluation Measures

##### Program Acceptability

Program acceptability, including satisfaction, usability, and appropriateness, was evaluated using a process evaluation survey developed for the study. Participants were asked to rate each of the components (quiz, goal setting, and creating strategies) as well as the website overall on a 5-point Likert scale from strongly agree (5) to strongly disagree (1) for usefulness, relevance, usability, and ability to motivate. Participants also rated their overall satisfaction with the EATS intervention, from very satisfied (5) to very unsatisfied (1); whether the intervention met their expectations, from strongly agree (5) to strongly disagree (1); and their opinion on the length of the intervention, from too much time (5) to too little time (1). The participants were also asked a series of open-ended questions relating to their likes and dislikes and suggestions for improvement for each of the intervention components and the intervention overall, as well as reasons for not visiting Web pages or using intervention components where relevant.

##### Intervention Usage

Intervention usage was objectively tracked using Google Analytics (Google LLC) and through the Qualtrics Survey Platform (Qualtrics). This included device used, website visit duration, number of pages viewed, and links accessed, including whether participants accessed the quiz, goal setting, and creating strategies components. For the goal setting and creating strategies components, the number of goals and strategies set was also recorded.

##### Recruitment and Retention

The number of individuals enquiring, screened for eligibility, consenting, randomized, completing the intervention, completing process evaluation, and completing follow-up were recorded.

#### Impact Evaluation Measures

##### Diet Quality

Dietary intake was assessed using the validated Australian Eating Survey Food Frequency Questionnaire (AES FFQ) [51], a self-administered 120-item semi quantitative FFQ, with participants being asked to report usual intake over the previous 3 months. Diet quality—Australian Recommended Food Score (ARFS)—was derived from FFQ responses. ARFS is calculated using a subset of 70 items from the AES FFQ relating to intake of fruits, vegetables, meat and flesh foods, nonmeat and flesh protein foods, breads and cereals, dairy foods, and water and spreads and sauces [52]. The ARFS is a summation of points scored for each item, with most items scoring 1 point for a consumption frequency of greater than or equal to once per week, and the total score ranging from 0 to 73 points. A higher ARFS reflects higher diet quality, including greater variety, more optimal nutrient intakes, and closer alignment with the Australian Dietary Guidelines [44].

##### Dietary Intake Related to the Target Eating Behaviors

Intake of fruits, vegetables, discretionary foods, and breakfast were determined from the AES FFQ. This included grams per day and the ARFS subscale for fruits and vegetables, percentage of daily energy intake for discretionary foods, and days per week for breakfast *(Never, 1-2 days, 3-4 days, 5 or more days).*

##### Alcohol Intake

Alcohol intake was assessed using 2 items from the New South Wales Adult Population Health Survey [35], including frequency of alcohol consumption and number of standard drinks usually consumed per drinking occasion.

##### Self-Efficacy for Performing Target Eating Behaviors

Self-efficacy for performing the target eating behaviors was assessed using questions derived from the Project EAT II Survey for Young Adults [53]. The participants were asked to rate their level of confidence on a scale of Not at all confident (1) to Very confident (4) for each behavior, for example, Eat at least 2 serves of fruit each day.

##### Quality of Life

Quality of life was assessed using the Quality of Life Enjoyment and Satisfaction Questionnaire short form [54]. The participants were asked to rate their satisfaction over the previous week on a scale of very good (5) to very poor (1) for each of the 14 items, for example, physical health.

##### Well-Being

Well-being was assessed using the World Health Organization-Five Well-being Index [55]. The participants were asked to rate how they were feeling over the previous 2 weeks on a scale of all of the time (5) to at no time (0) for each of the 5 statements, for example, I have felt cheerful and in good spirits.

#### Measures of Potential Contamination

Included in the follow-up survey were 3 items that assessed what, if anything, the participants had used to help them change their eating behavior as a means of assessing potential contamination of intervention effects. These included whether participants had made changes to their eating behavior over the previous 3 months *(Yes or No)*, and, if yes, an open-response item asking participants to describe the changes; and included a multiple-choice question asking participants to indicate what they had used to help them change behavior, for example, EATS, other research study, program—for example, Lite n’ Easy or Weight Watchers—and other.

### Data Analysis Plan

Data will be analyzed using Stata software version 14.1 (StataCorp LLC). Process evaluation data will be reported as means and SDs for quantitative questions, for example, scoring of individual program components such as usefulness of the goal-setting feature, and open question responses, such as suggested improvements for EATS, will be compiled and described qualitatively. Intervention usage will be assessed via analysis of usage metrics, including the time spent completing individual components and number of times the links were accessed, to allow comparison with other electronic health (eHealth) research studies. Impact evaluation, that is, to determine the efficacy of the EATS program compared with the control, will be determined using an intention-to-treat approach. All the results for all the participants who enter the study will be included in the analyses. Analyses will assess the difference between the intervention and control groups for the change from baseline to 3 months for all outcomes. Each study effect will be tested as the group by time interaction using a linear mixed model where time will be treated as a repeated measure. The effect sizes and 95% CIs will be reported for each outcome measure. A sensitivity analysis will be conducted to explore the impact of missing data on the primary impact evaluation measure (diet quality).

## Results

The pilot RCT has now been completed; the collection of the 3-month follow-up data was completed on July 12, 2018. Data analysis is currently being conducted.

## Discussion

### Principal Findings

This project used the PRECEDE-PROCEED model to develop a brief Web-based nutrition intervention for young adult university students and plan the evaluation. Nutrition interventions are needed in this target group because of the high rates of unhealthy eating behaviors and the known impacts of this on health and well-being and long-term chronic disease risk [56]. The difficulty is in engaging young adults because of the transitional nature of this life stage and the many factors influencing their health behaviors [9].

The pilot RCT will evaluate whether EATS is deemed acceptable and appropriate by young adult university students and whether it can improve diet quality over a 3-month follow-up period. The process evaluation will provide important feedback from the target population on the appropriateness of the mode of delivery, intervention duration, and individual intervention components. This will be utilized to update and modify the intervention, whereas the intervention usage data will be compared with other eHealth studies to assess participant engagement. Utilizing the university setting, as well as the eHealth mode of intervention delivery, shows promise as a means of effectively targeting and engaging young adults [14,57]. In addition, the development of EATS as a brief intervention draws on the precedence of successful brief interventions to reduce alcohol use among university students [20]. Therefore, the combination of a strong methodological approach to intervention development, the use of a setting with the potential for wide reach among young adults, and the use of an intervention approach with demonstrated efficacy suggest that the EATS intervention may be successful.

### Implications

If the evaluation results are positive, the potential implications will be significant in terms of translation and addressing the important health problem of poor diet among young adult university students. The long-term goal of this project is translation into University Health Promotion, that is, to implement EATS as a readily available program for all UON students, facilitated by the University Health Promotion staff. The key strategy to achieve this is the engagement of the steering committee, including key stakeholders to assist in translation. Furthermore, the chosen mode of delivery (ie, automated website) minimizes the ongoing resources required for program delivery. This paper adds to this evidence base and provides a detailed example for future researchers and health professionals working with this group and setting. If successful, there is also the potential that the intervention could be transferable to other universities nationally or internationally.

## Abbreviations

AES FFQ: Australian Eating Survey Food Frequency Questionnaire
ARFS: Australian Recommended Food Score
BCT: behavior change technique
EATS: Eating Advice To Students
eHealth: electronic health
RCT: randomized controlled trial
SCT: social cognitive theory
SHLS: Student Healthy Lifestyle Survey
TPB: theory of planned behavior
UON: University of Newcastle

## References

[1] (2016). Australian Bureau of Statistics.

[2] (2015). National Cancer Institute.

[3] Grech A, Allman-Farinelli M (2016). Prevalence and period trends of overweight and obesity in Australian young adults. Eur J Clin Nutr.

[4] Gordon-Larsen P, The NS, Adair LS (2010). Longitudinal trends in obesity in the United States from adolescence to the third decade of life. Obesity (Silver Spring).

[5] Nelson MC, Story M, Larson NI, Neumark-Sztainer D, Lytle LA (2008). Emerging adulthood and college-aged youth: an overlooked age for weight-related behavior change. Obesity (Silver Spring).

[6] Leonard A, Hutchesson M, Patterson A, Chalmers K, Collins C (2014). Recruitment and retention of young women into nutrition research studies: practical considerations. Trials.

[7] Lam E, Partridge SR, Allman-Farinelli M (2016). Strategies for successful recruitment of young adults to healthy lifestyle programmes for the prevention of weight gain: a systematic review. Obes Rev.

[8] Ashton LM, Morgan PJ, Hutchesson MJ, Rollo ME, Collins CE (2017). Feasibility and preliminary efficacy of the 'HEYMAN' healthy lifestyle program for young men: a pilot randomised controlled trial. Nutr J.

[9] Munt AE, Partridge SR, Allman-Farinelli M (2017). The barriers and enablers of healthy eating among young adults: a missing piece of the obesity puzzle: a scoping review. Obes Rev.

[10] Allman-Farinelli MA (2015). Nutrition promotion to prevent obesity in young adults. Healthcare (Basel).

[11] Lytle LA, Svetkey LP, Patrick K, Belle SH, Fernandez ID, Jakicic JM, Johnson KC, Olson CM, Tate DF, Wing R, Loria CM (2014). The EARLY trials: a consortium of studies targeting weight control in young adults. Transl Behav Med.

[12] Hayba N, Partridge SR, Nour MM, Grech A, Allman Farinelli M (2018). Effectiveness of lifestyle interventions for preventing harmful weight gain among young adults from lower socioeconomic status and ethnically diverse backgrounds: a systematic review. Obes Rev.

[13] Partridge SR, Juan SJ, McGeechan K, Bauman A, Allman-Farinelli M (2015). Poor quality of external validity reporting limits generalizability of overweight and/or obesity lifestyle prevention interventions in young adults: a systematic review. Obes Rev.

[14] Plotnikoff RC, Costigan SA, Williams RL, Hutchesson MJ, Kennedy SG, Robards SL, Allen J, Collins CE, Callister R, Germov J (2015). Effectiveness of interventions targeting physical activity, nutrition and healthy weight for university and college students: a systematic review and meta-analysis. Int J Behav Nutr Phys Act.

[15] (2016). National Center for Education Statistics.

[16] (2016). Eurostat.

[17] (2017). Australian Government Department of Education and Training.

[18] (2015). Okanagan charter.

[19] Visser PL, Hirsch JK (2014). Health behaviors among college students: the influence of future time perspective and basic psychological need satisfaction. Health Psychol Behav Med.

[20] Dotson KB, Dunn ME, Bowers CA (2015). Stand-alone personalized normative feedback for college student drinkers: a meta-analytic review, 2004 to 2014. PLoS One.

[21] Kattelmann KK, Bredbenner CB, White AA, Greene GW, Hoerr SL, Kidd T, Colby S, Horacek TM, Phillips BW, Koenings MM, Brown ON, Olfert MD, Shelnutt KP, Morrell JS (2014). The effects of Young Adults Eating and Active for Health (YEAH): a theory-based web-delivered intervention. J Nutr Educ Behav.

[22] Green LW, Kreuter MW, Deeds SG, Partridge KB, Bartlett E (1979). Health Education Planning: A Diagnostic Approach.

[23] Kattelmann KK, White AA, Greene GW, Byrd-Bredbenner C, Hoerr SL, Horacek TM, Kidd T, Colby S, Phillips BW, Koenings MM, Brown ON, Olfert M, Shelnutt KP, Morrell JS (2014). Development of Young Adults Eating and Active for Health (YEAH) internet-based intervention via a community-based participatory research model. J Nutr Educ Behav.

[24] Glanz K, Rimer BK, Viswanath K (2015). Health Behavior: Theory, Research, and Practice.

[25] Deliens T, Verhoeven H, De Bourdeaudhuij I, Huybrechts I, Mullie P, Clarys P, Deforche B (2018). Factors associated with fruit and vegetable and total fat intake in university students: a cross-sectional explanatory study. Nutr Diet.

[26] Thorpe MG, Kestin M, Riddell LJ, Keast RS, McNaughton SA (2014). Diet quality in young adults and its association with food-related behaviours. Public Health Nutr.

[27] Peltzer K, Pengpid S (2015). Correlates of healthy fruit and vegetable diet in students in low, middle and high income countries. Int J Public Health.

[28] El Ansari W, Stock C, Mikolajczyk RT (2012). Relationships between food consumption and living arrangements among university students in four European countries - a cross-sectional study. Nutr J.

[29] El Ansari W, Stock C, John J, Deeny P, Phillips C, Snelgrove S, Adetunji H, Hu X, Parke S, Stoate M, Mabhala A (2011). Health promoting behaviours and lifestyle characteristics of students at seven universities in the UK. Cent Eur J Public Health.

[30] Papadaki A, Hondros G, A Scott J, Kapsokefalou M (2007). Eating habits of university students living at, or away from home in Greece. Appetite.

[31] Tanton J, Dodd LJ, Woodfield L, Mabhala M (2015). Eating behaviours of British university Students: a cluster analysis on a neglected Issue. Adv Prev Med.

[32] Bemel JE, Brower C, Chischillie A, Shepherd J (2016). The impact of college student financial health on other dimensions of health. Am J Health Promot.

[33] Papier K, Ahmed F, Lee P, Wiseman J (2015). Stress and dietary behaviour among first-year university students in Australia: sex differences. Nutrition.

[34] Li KK, Concepcion RY, Lee H, Cardinal BJ, Ebbeck V, Woekel E, Readdy RT (2012). An examination of sex differences in relation to the eating habits and nutrient intakes of university students. J Nutr Educ Behav.

[35] (2014). Centre for Epidemiology and Evidence.

[36] Whatnall M, Patterson A, Hutchesson M (2018). Determinants of eating behaviours in Australian university students. Nutr Diet.

[37] Deliens T, Clarys P, De Bourdeaudhuij I, Deforche B (2014). Determinants of eating behaviour in university students: a qualitative study using focus group discussions. BMC Public Health.

[38] Hartman H, Wadsworth DP, Penny S, van Assema P, Page R (2013). Psychosocial determinants of fruit and vegetable consumption among students in a New Zealand university. Results of focus group interviews. Appetite.

[39] Lacaille LJ, Dauner KN, Krambeer RJ, Pedersen J (2011). Psychosocial and environmental determinants of eating behaviors, physical activity, and weight change among college students: a qualitative analysis. J Am Coll Health.

[40] Oosterveen E, Tzelepis F, Ashton L, Hutchesson MJ (2017). A systematic review of eHealth behavioral interventions targeting smoking, nutrition, alcohol, physical activity and/or obesity for young adults. Prev Med.

[41] Whatnall MC, Patterson AJ, Ashton LM, Hutchesson MJ (2018). Effectiveness of brief nutrition interventions on dietary behaviours in adults: a systematic review. Appetite.

[42] Bandura A (1985). Social Foundations of Thought and Action: A Social Cognitive Theory.

[43] Ajzen I (1991). The theory of planned behavior. Organ Behav Hum Decis Process.

[44] (2013). National Health and Medical Research Council.

[45] Hutchesson MJ, Callister R, Morgan PJ, Pranata I, Clarke ED, Skinner G, Ashton LM, Whatnall MC, Jones M, Oldmeadow C, Collins CE (2018). A targeted and tailored eHealth weight loss program for young women: the be positive be health randomized controlled trial. Healthcare (Basel).

[46] Cooke PA, Tully MA, Cupples ME, Gilliland AE, Gormley GJ (2013). A randomised control trial of experiential learning to promote physical activity. Educ Prim Care.

[47] Michie S, Richardson M, Johnston M, Abraham C, Francis J, Hardeman W, Eccles MP, Cane J, Wood CE (2013). The behavior change technique taxonomy (v1) of 93 hierarchically clustered techniques: building an international consensus for the reporting of behavior change interventions. Ann Behav Med.

[48] Michie S, Johnston M (2012). Theories and techniques of behaviour change: developing a cumulative science of behaviour change. Health Psychol Rev.

[49] Shanyinde M, Pickering RM, Weatherall M (2011). Questions asked and answered in pilot and feasibility randomized controlled trials. BMC Med Res Methodol.

[50] Kypri K, Hallett J, Howat P, McManus A, Maycock B, Bowe S, Horton NJ (2009). Randomized controlled trial of proactive web-based alcohol screening and brief intervention for university students. Arch Intern Med.

[51] Collins CE, Boggess MM, Watson JF, Guest M, Duncanson K, Pezdirc K, Rollo M, Hutchesson MJ, Burrows TL (2014). Reproducibility and comparative validity of a food frequency questionnaire for Australian adults. Clin Nutr.

[52] Collins CE, Burrows TL, Rollo ME, Boggess MM, Watson JF, Guest M, Duncanson K, Pezdirc K, Hutchesson MJ (2015). The comparative validity and reproducibility of a diet quality index for adults: the Australian recommended food score. Nutrients.

[53] Neumark-Sztainer DR, Wall MM, Haines JI, Story MT, Sherwood NE, van den Berg PA (2007). Shared risk and protective factors for overweight and disordered eating in adolescents. Am J Prev Med.

[54] Mick E, Faraone SV, Spencer T, Zhang HF, Biederman J (2008). Assessing the validity of the Quality of Life Enjoyment and Satisfaction Questionnaire Short Form in adults with ADHD. J Atten Disord.

[55] (1998). Psychiatric Research Unit. WHO Collaborating Centre in Mental Health.

[56] Liu K, Daviglus ML, Loria CM, Colangelo LA, Spring B, Moller AC, Lloyd-Jones DM (2012). Healthy lifestyle through young adulthood and the presence of low cardiovascular disease risk profile in middle age: the Coronary Artery Risk Development in (Young) Adults (CARDIA) study. Circulation.

[57] (2017). International Telecommunication Union.

